# Semi-supervised Learning for Weed and Crop Segmentation Using UAV Imagery

**DOI:** 10.3389/fpls.2022.927368

**Published:** 2022-07-01

**Authors:** Chunshi Nong, Xijian Fan, Junling Wang

**Affiliations:** ^1^College of Economics and Management, Nanjing University of Aeronautics and Astronautics, Nanjing, China; ^2^College of Information Science and Technology, Nanjing Forestry University, Nanjing, China

**Keywords:** weed mapping, semantic segmentation, semi-supervised learning, precision agriculture, crop recognition

## Abstract

Weed control has received great attention due to its significant influence on crop yield and food production. Accurate mapping of crop and weed is a prerequisite for the development of an automatic weed management system. In this paper, we propose a weed and crop segmentation method, SemiWeedNet, to accurately identify the weed with varying size in complex environment, where semi-supervised learning is employed to reduce the requirement of a large amount of labelled data. SemiWeedNet takes the labelled and unlabelled images into account when generating a unified semi-supervised architecture based on semantic segmentation model. A multiscale enhancement module is created by integrating the encoded feature with the selective kernel attention, to highlight the significant features of the weed and crop while alleviating the influence of complex background. To address the problem caused by the similarity and overlapping between crop and weed, an online hard example mining (OHEM) is introduced to refine the labelled data training. This forces the model to focus more on pixels that are not easily distinguished, and thus effectively improve the image segmentation. To further exploit the meaningful information of unlabelled data, consistency regularisation is introduced by maintaining the context consistency during training, making the representations robust to the varying environment. Comparative experiments are conducted on a publicly available dataset. The results show the SemiWeedNet outperforms the state-of-the-art methods, and its components have promising potential in improving segmentation.

## Introduction

Weeds are unwanted wild plants that grow naturally and spread rapidly, and tend to compete with crops for water, sunlight, fertiliser, soil nutrition, etc. (Hasan et al., 2021). In recent years, weeds are regarded to pose the most threat to crop growth and could have a serious negative impact on crop yield and food production (Harker and O’Donovan, 2013). Therefore, it is essential to deploy resources to monitor the growth of weeds and reduce weeds for healthy crop cultivation. There are two traditional strategies that are used to reduce the influence of weeds: mechanical weed control (e.g., mowing, mulching and tilling) and chemical weed control (i.e., using herbicides; Rakhmatulin et al., 2021). Both strategies have drawbacks. Mechanical weed control might lead to erosion, and the mechanical arm can easily damage the crop and harm beneficial organisms, e.g., earthworm and spiders, in the soil. Current chemical weed control relies on the traditional full-drench spraying without distinguishing between crops and weeds, where most herbicides hit the ground but some of them may drift away (Kudsk and Streibig, 2003). This could result in wastage of large volume of pesticides, high costs and pollution of soil and water.

Due to the increased cost of labour, more attention has been given to health and environmental issues, and the automation of weed control has become an effective solution. Such automation enables weeding with reduced labour costs, where selective spraying techniques are capable of significantly reducing the use of herbicides. The prerequisite of an automatic weed management system is to detect weeds accurately (Liu and Bruch, 2020). Machine vision using field or airborne cameras is an efficient means to accomplish this task. Abouzahir et al. (2021) employed classical hand-drafted descriptor, i.e., HOG to construct visual words, and used a neural network for weed and plant classification. Che’Ya et al. (2021) designed a classification model based on hyperspectral reflectance for recognising three types of weeds. Islam et al. (2021) used different machine learning (ML) methods, i.e., random forest, k-nearest neighbours, and support vector machine, to detect weeds in arial images, and shows the use of random forest achieves the best performance. The above-mentioned methods only focus on image-level classification of weeds.

To better implement the subsequent control of weeds, weed detection needs to locate the position and identify the boundary between crop and weed precisely, i.e., to generate a weed map. To this end, semantic segmentation can be applied to automatically segment the weeds and crop. With the rapid advance of ML and deep learning (DL), semantic segmentation based on ML and DL (Long et al., 2015; Ronneberger et al., 2015; Chen et al., 2017; Zhao et al., 2017) has become more widely used for mapping weeds. Lottes et al. (2017) proposed mapping weeds by including vegetation detection, plant-specific feature extraction and classification using RGB images acquired from a low-cost unmanned aerial vehicle (UAV). Castro et al. (2018) attempted to segment weeds using UAV imagery during the early growth stage of the crops. Alexandridis et al. (2017) designed four detection classifiers to distinguish *Silybum marianum* from other vegetation, where different types of features, i.e., three spectral bands and texture are extracted for the classifiers. However, traditional machine learning methods only capture low-level hand-crafted features, i.e., shape, texture, colour, etc., which tend to be not robust and lack generalization.

For DL based weed mapping, Sa et al. (2018) collected multispectral and RGB imagery covering 16,550 m^2^ sugar beet fields using a five-band RedEdge-M and a four-band Sequoia camera. Their method utilises a semantic segmentation model to distinguish the vegetarian from soil and improves its effectiveness *via* varying channels or their combinations. Compared to only using RGB channel, the model uses nine multispectral channels to achieve the best performance with AUC [i.e., area under the ROC (i.e., Receiver Operating Characteristic) curve of 0.839, 0.863, and 0.782 for background, crop, and weed, respectively]. Huang et al. (2018) applied full convolutional network (FCN) to generate weed distribution maps, where a fully connected conditional random field (CRF) is employed to enhance the spatial details. Experimental results show the method outperforms pixel-based support vector machine (SVM) and the traditional FCN-8 s in terms of mean Intersection-over-Union (IoU) and accuracy. Ramirez et al. (2020) proposed a weed segmentation framework based on DeepLabv3 architecture using an aerial image. They demonstrated that increasing the balance of data and enhancing the spatial information resulted in better performance in terms of AUC and F1-score. Ma et al. (2019) constructed a semantic segmentation method based on FCN to distinguish weed from rice seedlings with promising accuracy in segmenting weed, rice seedlings, and soil background. You et al. (2020) proposed a weed/crop segmentation model based on deep neural network (DNN), which integrates four additional modules, i.e., hybrid dilated convolution and dropblock, universal function approximation block, attention block, and spatial pyramid refined block. The performance of the model on two publicly available datasets is better than the state-of-art segmentation methods. However, all of the above-mentioned methods adopt fully supervised semantic segmentation networks, which require large amount of pixel-wise annotated data and are thus labour intensive. Although data augmentation techniques (i.e., image rotation, cropping, flipping, etc.) are used to alleviate the problem of insufficient training data, the methods still need hundreds of pixel-wise annotated images for training an optimal model. In addition, due to the severe overlapping of weeds and crop in the field, it is not trivial to annotate the weed and crop pixel by pixel.

Compared with collecting annotated data that is time-consuming and labour-intensive, unannotated data are much easier to acquire. In addition, semi-supervised learning can make full use of the rich information in unannotated data, which significantly alleviates the workload of annotating images while retaining accuracy. Therefore, such an approach offers effective solution for mapping crop and weeds. To the best of our knowledge, there are few studies working on semi-supervised weed and crop mapping or classification. Pérez-Ortiz et al. (2015) proposed a weed mapping system using multispectral images acquired from UAV, which involves computing different vegetation indices, and row detection *via* Hough transform. They used different machine learning paradigms to achieve the best performance. However, their system is not end-to-end, and is not suitable for generalization due to the manually adjusted parameters used. Lottes and Stachniss (2017) proposed an online crop/weed mapping method by integrating vision-based classification and geometry-based classification, achieving a classification performance with an accuracy of greater than 95% in two sugar beet fields. However, these two methods are based on traditional machine learning, which is not end-to-end, and heavily rely on feature extraction and classifier design. This is prone to error and could lead to the poor generalization. Jiang et al. (2020) proposed a model based on graph convolutional network to classify multi-species crops and weeds, by exploiting both labelled and unlabelled image features. Khan et al. (2021) used generative adversarial network to augment the training samples, enhancing the capability in distinguishing crop from weeds in UAV imagery. Nevertheless, both methods only focus on exploiting semi-supervised learning for image-level classification of crop and weeds, not tackling the pixel-wise mapping problem.

Unlike image-level classification, pixel-wise crop/weed segmentation is much more challenging due to two essential characterises exclusively existing in crop and weed field. First, weeds tend to grow disorderly and might spread amongst crop plants, which may lead to overlapping and occlusions. Second, there exists the ambiguity in weed/crop mapping, where it could be difficult to distinguish the crop from the background as they share the similarity in colour and texture. Furthermore, UAV is a popular means for monitoring farmland and mapping the crop and weeds, as they are flexible, cost-saving, easily manipulated and do not affect the fields through soil compaction as ground vehicles do. Therefore, we focus on the weed and crop mapping using UAV imagery, which brings an additional challenge, namely the size of crop and weed is smaller in these images.

In this paper, we aim at exploring the problem of crop and weed mapping using UAV imagery and propose a semi-supervised segmentation framework for segmenting weeds and crop in order to significantly reduce the requirement of manually annotated data. To address the challenges in crop/weed segmentation using semi-supervised learning, the proposed method uses an attention strategy by integrating it to encoded feature from the encoder of the segmentation model to generate the attention enhanced feature. The enhanced feature provides useful information of the targets, i.e., crop and weeds, and highlight the target feature while mitigating the impact of background. To avoid the ambiguity caused by the similarity between crop and weeds, we employ online hard example mining (OHEM) to separate the regions that are easily confused by refining the positive samples with low confidence. In summary, the proposed method automatically segments the weeds, crop and soil (background) accurately, where semi-supervised learning greatly reduces the cost of labour and the training time.

The main contributions of our work are:

1. An efficient semi-supervised semantic segmentation model, specifically for crop and weed mapping using UAV optical imagery. To the best of our knowledge, we are the first to address the challenges exclusively existing in crop/weed mapping based on semi-supervised learning.
2. A multiscale enhanced feature by integrating the selective kernel attention with the encoded features, highlighting the significant features of the target crop and weeds, and further increasing the ability to identify the weed/crop in varying scales in UAV images.
3. OHEM for focusing more on those pixels that not easily distinguishable, effectively reducing inaccurate segmentation caused by the similarity and overlapping between crop and weeds.

The remainder of the paper is structured as follows: The proposed method and dataset are presented in detail in section Proposed Method and Data. Section Results and Discussion discusses the implementation setting, experimental results, and comparative analysis. The conclusions drawn are presented in section Conclusion and Future Work.

## Proposed Method and Data

**Figure 1:**
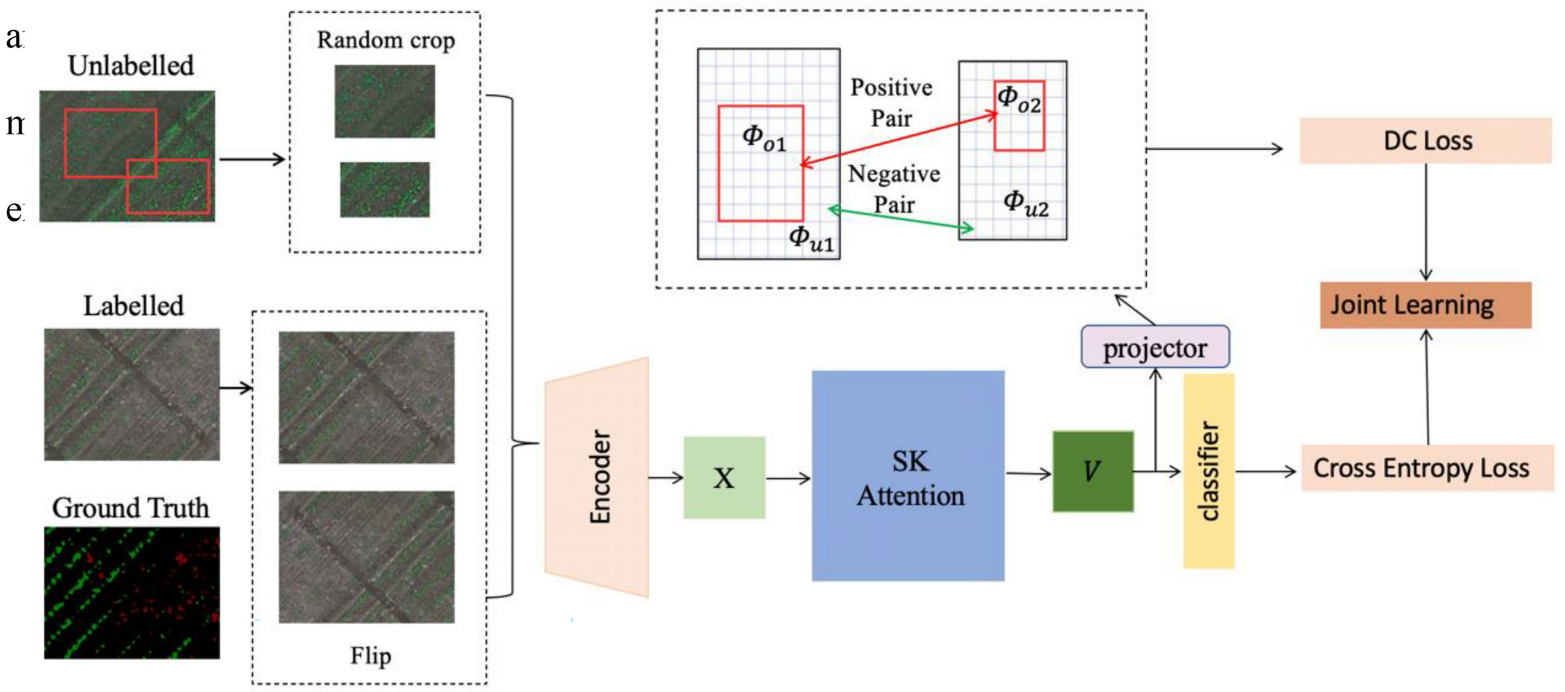
Overall framework of the proposed method, SemiWeedNet.

This section provides the details of the proposed method including the encoder, attention module, and the joint loss for supervised and unsupervised learning. The overall framework of the proposed method, SemiWeedNet, is shown in Figure 1. The section also presents the data used in our experiments to evaluate the performance of the proposed method.

### Semi-supervised Method for Crop/Weed Segmentation

#### DeeplabV3+ Architecture

**Figure 2:**
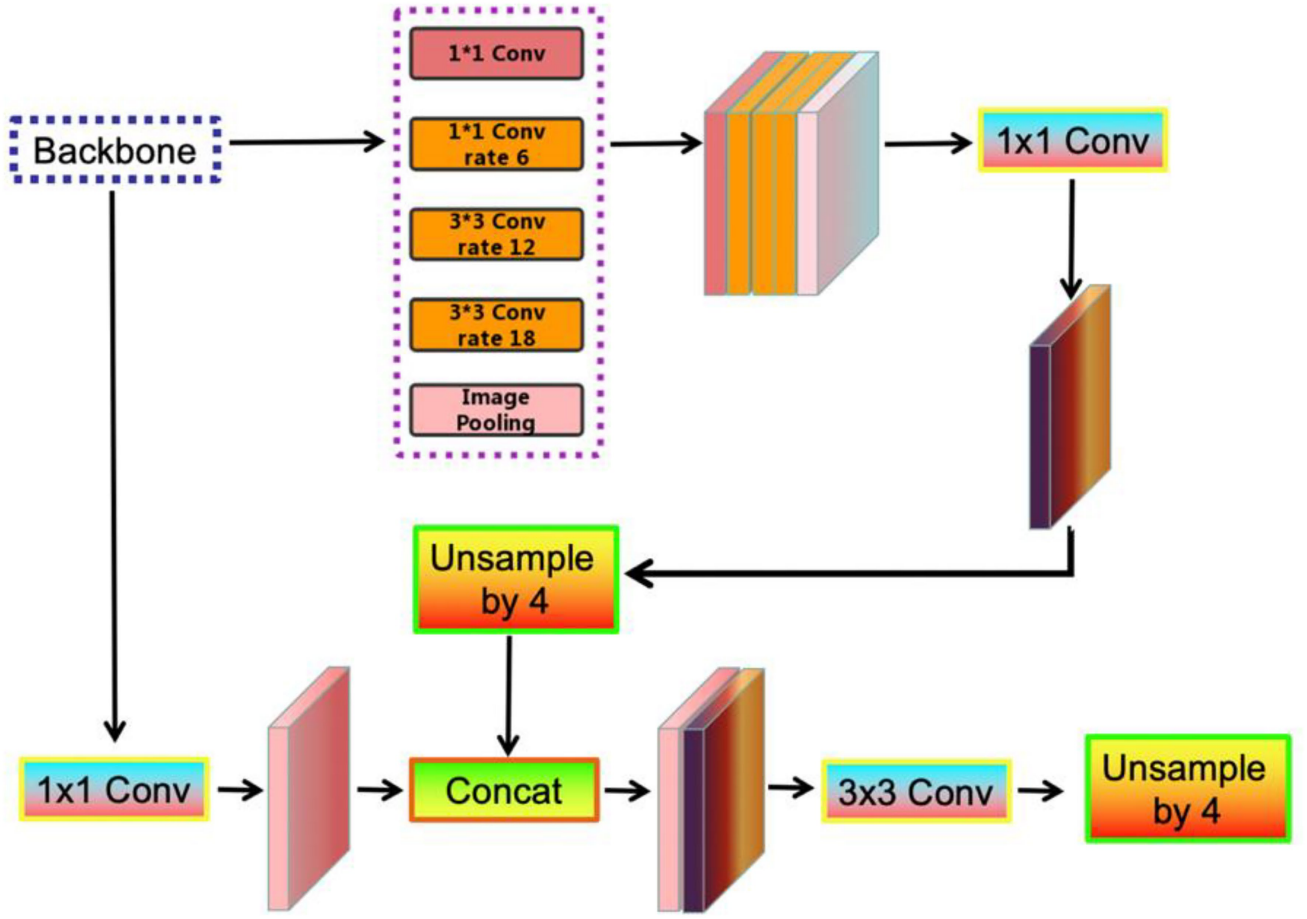
The architecture of DeepLabV3+, where 1 × 1 Conv and 3 × 3 Conv denote the convolution with the kernel size of 1 × 1 and 3 × 3, respectively, Unsample denotes the bilinear upsampling operation, and Concat denotes the concatenation of feature.

The DeepLab series network was originally proposed by Chen et al. (2014), which addresses the poor localization characteristic of deep network by integrating feature from the final network layer with a fully connected CRF. The DeepLabV3 network (Chen et al., 2017) incorporates atrous convolution modules and an augmented atrous spatial pyramid pooling (ASPP), discarding the CRF, to enhance the capability of extracting multi-scale information and encoding the global structure information. To locate sharper object boundary, DeepLabv3+ (Chen et al., 2018) as shown in Figure 2 extends DeepLabV3 by integrating an effective decoder to refine the results.

In addition, Xception model are employed in DeepLabv3+, where depth-wise separable convolution is applied to replace the convolutional layers in ASPP and decoder. In this paper, we employ DeepLabV3+ as our basic encode-decode framework due to its two competitive advantages: (1) Enabling to depict the multiscale feature that is widely existing in crop/weed maps; and (2) Significantly reducing the computational complexity, which is appropriate for field monitoring.

DeepLabV3+ comprises an encoder and a decoder. The input image for the encoder is extracted by the depth-separable convolutional layers of the different channels in the backbone model. The extracted feature maps are then processed by the ASPP module and the channel attention (CA) module. This is followed by 1 × 1 convolution, where the atrous convolution with an atrous stride of 6, 12, and 18 and the global average pooling are used for stitching. The CA module is then used to fuse the feature maps obtained from the ASPP module, where the 1 × 1 depth separable convolution is used in the CA module to reduce the dimensionality. The final features containing 256 channels, extract rich contextual information and effectively capture high-level semantics.

The feature maps extracted from the encoder are first bilinearly up-sampled by a factor of 4, and simultaneously concatenated with the corresponding low-level features from the backbone with the same spatial resolution. An additional 1 × 1 convolution is applied to the low-level features to decrease the dimensionality of the channel. A 3 × 3 convolution is applied to the features and followed by another simple bilinear up-sampling. The features are then gradually refined to recover spatial information and are used to generate the final segmentation results.

#### Multiscale Enhancement Module

**Figure 3:**
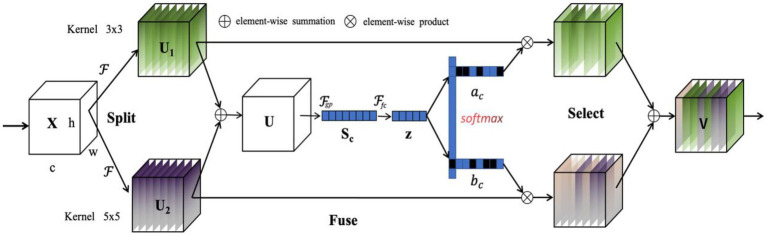
Selective kernel attention enhancement module.

Although the DeepLabv3+ model aggregates multiscale features, its convolution kernel size is fixed and thus is insufficient for our scenarios due to the high variability of the targets (i.e., crop and weeds) and complex background. Based on our observation, the traditional DeepLabV3+ encoder–decoder module sometimes fails to identify the entire regions of weeds and crop, especially in some small size areas, leading to a highly incorrect segmentation. To solve the problem, we design a multiscale feature enhancement module (MFEM) by integrating the effective attention mechanism to the encoded feature, where Selective Kernel Attention (SKA) is exploited due to its computational efficiency. SKA extracts the different size of the convolutional kernels by combining squeeze-excitation module with multi-scale information, where the features extracted using different kernel size are refined and thus achieve better representation. SKA consists of three parts: Split, Fuse, and Select as illustrated in Figure 3. The Split operator generates multiple paths with various kernel sizes based on different sized receptive fields of neurons. The Fuse part then combines the information of multiple paths to acquire a more comprehensive representation for selection weights. The Select part aggregates the feature maps of kernels with varying size based on the selection weights.

Specifically, given a feature map $X\in {\mathbb{R}}^{H^{\prime }\times W^{\prime }\times C^{\prime }}$ , we perform the mapping by applying two convolution operations with the kernel size of 3 and 5 as


(1) $$\begin{array}{c} F_{1}:X\to U_{1}\in R^{H\times W\times C}\\ F_{2}:X\to U_{2}\in R^{H\times W\times C} \end{array}\;$$

where *H*, *W,* and *C,* respectively, denote the height, width, and number of channels for feature maps. $F_{1}$ and $F_{2}$ comprise depthwise convolutions, Batch Normalisation (BN; Ioffe and Szegedy, 2015) and ReLU (Nair and Hinton, 2010) activation.

After the Split part, the Fuse part fuses the two mapped features *via* element-wise summation, which is capable of better enhancing the global structure information while retaining the local details in crop images. The module consists of four sub-modules: split, fuse, and scale, i.e.,


(2) $$\;\;\;U=U_{1}+U_{2}$$

where the fused feature maps U combine the feature information both from $U_{1}$ and $U_{2}$ . The feature maps are then embedded in channel-wise statistics $S\in {\mathbb{R}}^{C}$ *via* global average pooling, where the *c*-th element of S is computed by compressing the spatial information of U , i.e.,


(3) $$S_{c}=F_{\mathrm{gp}}\left(U_{c}\right)=\frac{1}{H\times W}\overset{H}{\underset{i=1}{\sum }}\overset{W}{\underset{j=1}{\sum }}U_{c}\left(i,j\right)$$

To promote the meaningful feature and suppress un-informative one, a simple fully connected (FC) layer is applied to reduce the dimensionality, followed by the BN and ReLU. The resultant feature descriptor is defined as


(4) $$\;z=F_{FC}\left(S\right)=\delta \left(B\left(WS\right)\right)$$

where B denotes the BN operation, δ denotes the ReLU function, and $W\in R^{d\times C}$ . We use a reduction ratio *r* to control the value of *d*, i.e.,


(5) $$d=\max\left(\frac{C}{r},L\right)$$

where L denotes the minimal value of *d*.

In the Select part, a soft attention across channels is exploited to adaptively select different spatial scales of information, which is supervised by the feature descriptor **z**. A softmax operator is applied on the channel-wise digits, i.e.,


(6) $$a_{c}=\frac{e^{A_{c}z}}{e^{A_{c}z}+e^{B_{c}z}},\;\;b_{c}=\frac{e^{B_{c}z}}{e^{A_{c}z}+e^{B_{c}z}}$$

where $A,B\in R^{C\times d},$ and *a* and *b,* respectively, denote the soft attention vector for $U_{1}\;$ and $U_{2}$ . Here, $A_{c}\in R^{1\times d}$ is the *c-*th row of A and $a_{c}$ is the *c-*th elements of a. Similarly, for $B_{c}$ and $b_{c}$ . The final feature map **V** is computed *via* the attention weights on various kernels, i.e.,


(7) $$V=a_{c}\cdot U_{1}+b_{c}\cdot U_{2},\;\;\;a_{c}+b_{c}=1$$

where $V=\left[V_{1},V_{2}\ldots ,V_{c}\right]$ .

The proposed MFEM effectively achieves multi-scale information existing in crop/weed segmentation by adaptively adjusting the respective field sizes, which significantly improves the performance of segmentation in the field.

#### Consistency Regularisation for Unsupervised Learning

There are two batches of inputs, $x_{l}$ and $x_{u}$ , respectively denoting labelled and unlabelled data. As for the general semantic segmentation, the encoder architecture E embeds the labelled image in the feature maps $f_{l}=E\left(x_{l}\right)$ , and the decoder makes predictions $p_{l}=C\left(f_{l}\right)$ . The learning process is provided by ground truth labels $y_{l}$ using the standard cross entropy loss $L_{ce}$ . With respect to an unlabelled image, we randomly crop two patches $x_{u1}$ and $x_{u2}$ with an overlapping region $x_{o}$ , and then augment $x_{u1}\;$ and $x_{u2}$ using low-level augmentation. The two augmented patches are then fed to the encoder model E to obtain the feature map $f_{u1}$ and $f_{u2}$ , respectively. Following the work in (Chen et al., 2020), the obtained two features are embedded using nonlinear projection as Φ, i.e.,


(8) $$\phi_{u1}=\Phi \left(f_{u1}\right)$$

(9) $$\;\;\;\phi_{u2}=\Phi \left(f_{u2}\right)$$

Accordingly, the features from the overlapping areas in $\phi_{u1}$ and $\phi_{u2}$ are referred as $\phi_{o1}$ and $\phi_{o2}$ , respectively, where the $\phi_{o1}$ and $\phi_{o2}$ should remain consistent under different contexts.

To this end, we use a context-ware consistency constraint, i.e., Directional Contrastive (DC) Loss, to enable the features from the overlapping areas to remain consistent with each other. The DC loss is inspired by the contrastive loss, which pulls the positive samples closer while separating the negative samples belonging to other classes. In our case, the features from overlapping locations $\phi_{u1}$ and $\phi_{u2}$ are regarded as a positive pair as they share the same pixels despite under different contexts, and any two features in $\phi_{u1}$ and $\phi_{u2}$ from different locations are regarded as a negative pair. Unlike contrastive loss, the DC loss further exploits a directional alignment for the positive pairs, which effectively avoids the high confident feature from suppressing the low confident one. This is because the prediction with higher confidence tends to be more accurate, and the feature with lower confidence need to be aligned to its higher confident counterpart. The confidence of each feature $\phi_{u1}$ is measured using maximum probability among all classes, i.e., $\max\left(C\left(f_{i}\right)\right)$ . For the *t-*th unlabelled image, the DC loss $L_{dc}^{\;t}$ is computed as


(10) $$\begin{array}{c} l_{dc}^{t}\left(\phi_{o1},\phi_{o2}\right)=\\ -\frac{1}{N}\underset{h,w}{\sum }M_{d}^{h,w}\cdot \log\frac{r\left(\phi_{o1}^{h,w},\phi_{o2}^{h,w}\right)}{r\left(\phi_{o1}^{h,w},\phi_{o2}^{h,w}\right)+\sum_{\phi_{n}\in F_{u}}r\left(\phi_{o1}^{h,w},\phi_{n}\right)} \end{array}$$

(11) $$M_{d}^{h,w}=1\left\{\max C\left(f_{o1}^{h,w}\right)<\max C\left(f_{o2}^{h,w}\right)\right\}$$

(12) $$L_{dc}^{\;t}=l_{dc}^{t}\left(\phi_{o1},\phi_{o2}\right)+l_{dc}^{t}\left(\phi_{o2},\phi_{o1}\right)$$

where $l_{dc}^{t}$ denotes the loss between the features at the two locations $\phi_{o1}$ and $\phi_{o2}$ , *N* is the number of spatial locations of overlapping area, *h* and *w* represent the 2-D spatial locations, $\phi_{n}$ denotes negative counterpart of the feature $\phi_{o1}^{h,w}$ and r represents the exponential function of the cosine similarity *s* between two features with a temperature τ , i.e., $r\left(\phi_{1},\phi_{2}\right)=\exp\left(s\left(\phi_{1},\phi_{2}\right)/\tau \right)$ , and $F_{u}$ denotes the set of negative samples. Since more negative samples result in better performance, a memory bank is used to store the features from the last few batches to acquire more negative samples (Lai et al., 2021). The final loss is then computed by summing the loss of each image, i.e.,


(13) $$L_{dc}=\frac{1}{T}\overset{T}{\underset{t=1}{\sum }}L_{dc}^{\;t}$$

where *T* denotes the batch size during training.

#### Loss Function With OHEM Strategy

The joint loss function of the proposed semi-supervised based method comprises two parts: cross entropy loss $L_{ce}\;$ for supervised learning, and consistency constraint loss $L_{dc}$ for unsupervised learning, which is defined as


(14) $$L=L_{ce}+\lambda L_{dc}$$

where λ is the hypermeter that balances the supervised loss and the unsupervised loss.

Based on our observation on samples, there are two problems that we need to address. First, the samples of different classes, i.e., crop, weeds and soil are imbalanced, leading to inefficient training. This is because the model may focus more on the samples that can be easily learned and ignore those samples that are difficult to be distinguished, degrading the model performance. Second, the ambiguous boundary of crop and weed due to overlapping and occlusion makes it more difficult for the model to identify the targets. The standard cross entropy loss could not handle these two problems. Thus, we employ OHEM to refine the training of the model, which focuses on those samples, which are difficult for the model to predict during training. The OHEM is first used to filter the input pixels, where pixels that are difficult to predict with a high impact on classification are selected for training in stochastic gradient descent (Shrivastava et al., 2016). Specifically, we modify the loss layer to select the difficult examples, where the loss for all pixels is computed, and is then sorted to select the difficult pixels. The nondifficult pixels are finally set to 0, and hence no gradient updates. The OHEM effectively deals with the problem of difficult samples existing in crop/weed mapping, which lead to better training, and thus increases the performance in segmentation.

### Dataset

**Figure 4:**
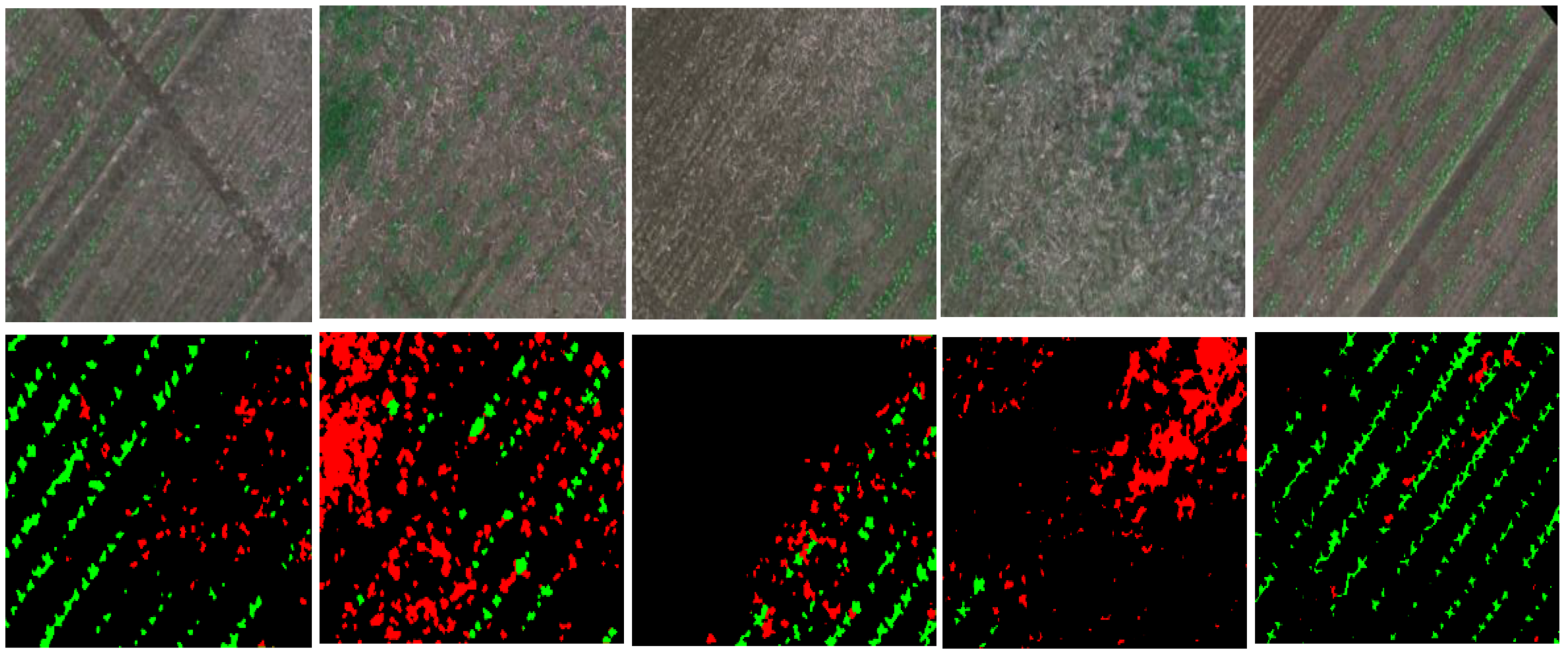
Image samples with ground truth mask from WeedMap. Green denotes the crop, Red denotes the weed, and Black denotes the background (soil).

To evaluate the effectiveness of the proposed semi-supervised learning segmentation method for crop/weed mapping, we use a publicly available dataset WeedMap (Sa et al., 2018) to conduct experiments. This dataset is collected from two sugar fields in Switzerland and Germany using two UAV platforms mounting two multispectral sensors, i.e., RedEdge and Sequoia. The platforms include Orthomosaic and Tile folders which, respectively, generate orthostatic maps and the associated tiles at a fixed size of 480 × 360. There are seven subsets of images denoting the different parts of the fields, where the subsets numbered from 000 to 004 are acquired by RedEdge in Germany, and those numbered from 005 to 007 are acquired by Sequoia in Switzerland. These images are used to generate tile images from an orthostatic map by using a sliding window, where some tiles may contain invalid pixel values. In our experiment, we select the effective tile images that contain no invalid pixels, and only choose the RGB channel as the input of our model. Overall, 289 RGB pixel-wise labelled images are collected from the subfolders of 000 to 004 (as shown in Figure 4). These images are randomly split into training set and testing set in the ratio of 8:2.

## Results and Discussion

In this section, the implementation details are demonstrated, the segmentation results are compared with state-of-the-art methods qualitatively and quantitatively. This section also presents the ablation study to evaluate the contributions of the various elements of the proposed method.

### Implementation Details

DeepLabV3+ is employed as the encoder–decoder network of the proposed method, SemiWeedNet, due to its effectiveness on multi-scale information, where Resnet50 and Resnet101 are used as the backbone. Since other existing state-of-the-art methods adopt Resnet as the backbone, we replace Inception model with the Resnet in our experiment for fair comparison.

The proposed method is implemented using Pytorch toolbox on a workstation with an NVIDIA RTX3080Ti GPU. The input images are resized to 480 × 480 pixels, and then augmented using random flipping. During training, we use SGD optimizer and set the learning rate, weight decay, and momentum to 0.02, 0.0001, and 0.9, respectively. The training batch size is set to 8, including 4 labelled and 4 unlabeled images. The weight λ for unsupervised loss is set to 0.7.

The Intersection-over-Union (IoU) for each class and mean Intersection-over-Union (mIOU) are employed as our evaluation metrics. IoU is also known as the Jaccard Index, and is a statistic indicating the similarity and diversity of samples. In semantic segmentation, IoU denotes the ratio of the intersection of the pixel-wise classification results and the ground truth, to determine the spatial overlap between the prediction and ground truth, i.e.,


(15) $$\mathrm{IoU}=\frac{n_{ii}}{t_{i}+\sum_{j}n_{ji}+n_{ii}},\;\;\;j\neq i$$

where $n_{ii}$ denotes the total number of pixels both predicted and labelled as class *I*, and $n_{ij}$ denotes the number of pixels of class *i*-th predicted to belong to class *j*, and $t_{i}$ is the total number of pixels of class *i*th in ground truth segmentation. The mIoU is computed by averaging the IoU of all classes, i.e.,


(16) $$mIoU=\frac{1}{k}\overset{k}{\underset{j=1}{\sum }}\frac{n_{jj}}{t_{i}+\sum_{j}n_{ij}+n_{ii}},i\neq j$$

### Performance of SemiWeedNet and Analysis

To evaluate the effectiveness of SemiWeedNet, we made comparisons with state-of-the-art methods including CAC (Lai et al., 2021), ST++ (Yang et al., 2021), Adv-Semi (Hung et al., 2018) and cycleGAN (Zhu et al., 2017). We implemented these methods within a unified framework following their official code, where the same base backbone (i.e., Resnet) is used and the same data lists are used for training and testing. We compared the proposed method under the setting with various labelled data proportions, i.e., 2/8, 3/7, 5/5 and full labelled data. In the full data setting, images fed to the unsupervised branch are simply collected from the labelled set.

**Figure 5:**
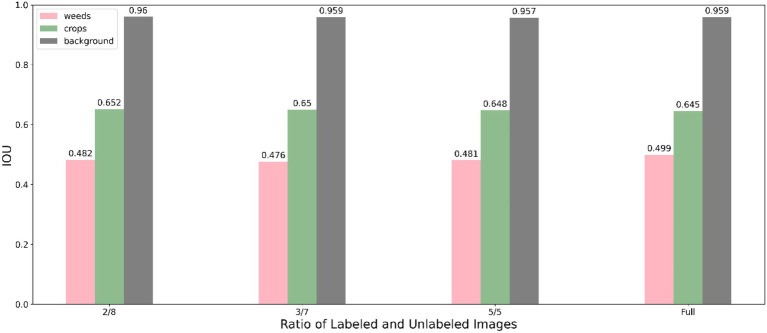
Performance of the proposed method using Resnet 50 under different labelled data proportions.

**Figure 6:**
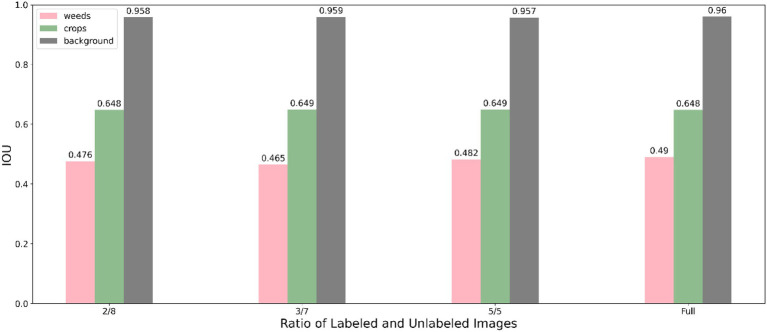
Performance of the proposed method using Resnet 101 under different labelled data proportions.

The segmentation performance of individual class using our method under various data proportions are shown in Figure 5 (using Resnet50 backbone) and Figure 6 (using Resnet101 backbone). We used only 20% labelled images incorporating unlabelled images and achieve a competitive performance with training using full labelled data, which significantly reduces the demand for annotating images.

In addition, we conducted experiments to compare the proposed method with other methods, and the results are shown in Table 1. The table shows that the segmentation result of proposed method outperforms other methods by a large margin on all data proportions. This is due to the facts that the proposed method uses the effective attention module to enhance the ability of capture the weed and crop with different scales. Furthermore, the online hard sample mining addresses the problem of overlapping between crop and weed. Both Adv-Semi and cycleGAN suffer from unstable training due to the use of adversarial learning, achieving unsatisfactory performances in our scenarios. ST++ and CAC use pseudo label based self-training method, which might lead to incorrect labeling especially in images with overlap and occlusion.

**Table 1:** Comparison with the baseline (SupOnly, i.e., using only supervised loss) and other state-of-the-art on WeedMap dataset with 2/8, 3/7, 5/5, and full labelled data.

Method	Backbone	2/8	3/7	5/5	full
SupOnly	Resnet50	0.664	0.675	**0.698**	0.700
CAC	Resnet50	0.663	0.673	0.679	0.676
ST++	Resnet50	0.598	0.597	0.598	0.613
Ours	Resnet50	**0.698**	**0.695**	0.695	**0.701**
SupOnly	Resnet101	0.670	0.673	0.688	0.705
CAC	Resnet101	0.675	0.679	0.683	0.686
ST++	Resnet101	0.599	0.607	0.594	0.611
Adv-Semi	Resnet101	0.599	0.586	0.587	0.622
cycleGAN	Resnet101	0.388	0.443	0.421	0.558
Ours	Resnet101	**0.692**	**0.690**	**0.696**	**0.700**

Values in bold denotes the best IoU performance.

**Figure 7:**
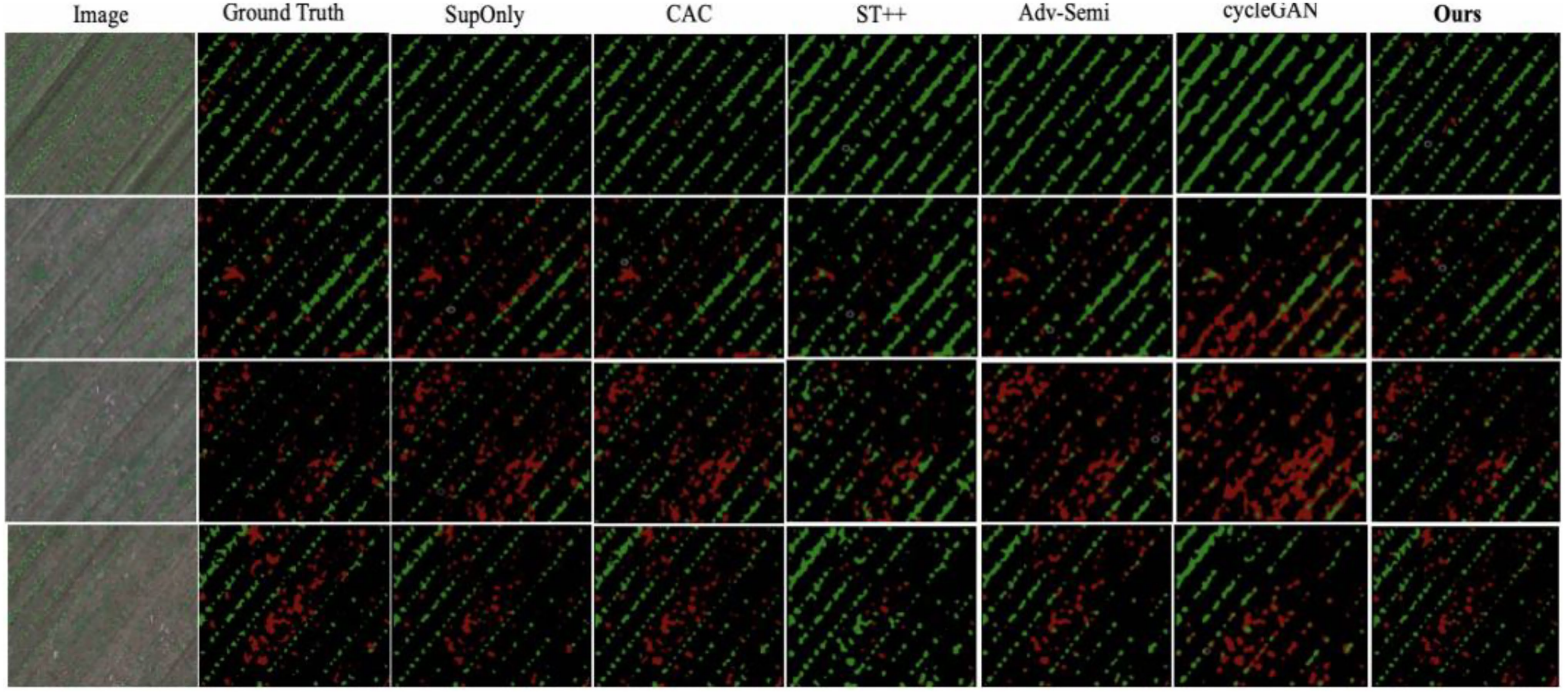
Visual comparison between our method with state-of-the-art methods and SupOnly.

We also present a visual comparison with other state-of-the-art methods in Figure 7. The figure shows that the proposed method is the only method which effectively identifies the crop and weeds with small size, and the results are almost consistent with the ground truth.

### Performance of SemiWeedNet Variants and Analysis

To thoroughly assess the performance of SemiWeedNet, we conducted an ablation study to illustrate the contribution of its key modules, and the results are shown in Table 2. We used DeepLabV3+ with Resnet101 as the segmentation network, and the baseline method, i.e., the model trained without using SKA enhancement and OHEM. We performed four sets of experiments: (1) Using baseline method; (2) Using SKA enhanced features; (3) Using OHEM; and (4) Using SemiWeedNet. Table 2 shows that SemiWeedNet yields a constant improvement under different data proportions, where both SKA enhancement and using OHEM have generally improved the performance for segmenting crop and weeds using UAV imagery. This verifies the effectiveness of the attention mechanism and hard sample mining strategy.

**Table 2:** Ablation study under different labelled data proportion.

Allocation strategy	Baseline	SK Attention	OHEM Loss	mIOU
2/8	✓			0.675
	✓	✓		0.690
	✓		✓	0.680
	✓	✓	✓	0.692
3/7	✓			0.679
	✓	✓		0.681
	✓		✓	0.686
	✓	✓	✓	0.690
5/5	✓			0.683
	✓	✓		0.695
	✓		✓	0.686
	✓	✓	✓	0.696
full	✓			0.686
	✓	✓		0.696
	✓		✓	0.693
	✓	✓	✓	0.700

## Conclusion and Future Work

In this paper, we focus on addressing the problem of automatic mapping crop and weeds using UAV acquired images from the real field environment, and propose a semi-supervised based semantic segmentation method, which significantly reduces the workload of manual annotations. Due to the complexity of the application environment, the multiscale enhancement module is designed by intergrading an effective attention mechanism to the encoded features to highlight the useful features of the targets, i.e., crop and weeds, while mitigating the influence of the background. OHEM is employed in the training of the model, which aims at addressing the similarity and overlapping of crop and weeds which resulted in poor recognition performance. An auxiliary consistency constraint is further introduced to fully exploit the information of the large amount of unlabelled images, to extract the meaningful and discriminative features for crop and weed segmentation. The performance of the proposed method is evaluated using WeedMap dataset, which demonstrates the superiority of our method compared with state-of-art methods and also shows the promising potential of our deigned modules. In the future, interesting possible extensions of this work could be designing a lightweight model by reducing the parameters and increasing the inference speed.

## Data Availability Statement

The original contributions presented in the study are included in the article/supplementary material, further inquiries can be directed to the corresponding author.

## Author Contributions

CN contributed to draft writing and editing. XF contributed to method design, supervision, and experiments design. JW contributed to visualisation. All authors contributed to the article and approved the submitted version.

## Funding

The work was supported by the Joint fund of Science & Technology Department of Liaoning Province and State Key Laboratory of Robotics, China (Grant No. 2020-KF-22-04).

## Conflict of Interest

The authors declare that the research was conducted in the absence of any commercial or financial relationships that could be construed as a potential conflict of interest.

## Publisher’s Note

All claims expressed in this article are solely those of the authors and do not necessarily represent those of their affiliated organizations, or those of the publisher, the editors and the reviewers. Any product that may be evaluated in this article, or claim that may be made by its manufacturer, is not guaranteed or endorsed by the publisher.

## References

[ref1] Abouzahir S. Sadik M. Sabir E. (2021). Bag-of-visual-words-augmented histogram of oriented gradients for efficient weed detection. Biosyst. Eng. 202, 179–194. doi: 10.1016/j.biosystemseng.2020.11.005

[ref2] Alexandridis T. K. Tamouridou A. A. Pantazi X. E. Lagopodi A. L. Kashefi J. Ovakoglou G.. (2017). Novelty detection classifiers in weed mapping: *Silybum marianum* detection on UAV multispectral images. Sensors 17:2007. 10.3390/s17092007 PMC5621143 28862663

[ref3] Castro A. Torres-Sánchez J. Peña J. Jiménez-Brenes F. Csillik O. López-Granados F. (2018). An automatic random Forest-obia algorithm for early weed mapping between and within crop rows using UAV imagery. Remote Sens. (Basel) 10:285. doi: 10.3390/rs10020285

[ref4] Che’Ya N. N. Dunwoody E. Gupta M. (2021). Assessment of weed classification using Hyperspectral reflectance and optimal multispectral UAV imagery. Agronomy 11:1435. doi: 10.3390/agronomy11071435

[ref5] Chen T. Kornblith S. Norouzi M. Hinton G. (2020). A simple framework for contrastive learning of visual representations. Int. Conf. Mach. Learn. 2020, 1597–1607.

[ref6] Chen L. C. Papandreou G. Kokkinos I. Murphy K. Yuille A. L. (2014). Semantic image segmentation with deep convolutional nets and fully connected crfs. arXiv [Preprint] arXiv:1412.7062. 10.1109/TPAMI.2017.2699184 28463186

[ref7] Chen L. C. Papandreou G. Schroff F. Adam H. (2017). Rethinking atrous convolution for semantic image segmentation. arXiv [Preprint] arXiv:1706.05587.

[ref8] Chen L. C. Zhu Y. Papandreou G. Schroff F. Adam H. (2018). Encoder-decoder with atrous separable convolution for semantic image segmentation. Proc. Eur. Conf. Comput.Vision 2018, 801–818. doi: 10.1007/978-3-030-01234-2_49

[ref9] Harker K. N. O'Donovan J. T. (2013). Recent weed control, weed management, and integrated weed management. Weed Technol. 27, 1–11. doi: 10.1614/WT-D-12-00109.1

[ref10] Hasan A. M. Sohel F. Diepeveen D. Laga H. Jones M. G. (2021). A survey of deep learning techniques for weed detection from images. Comput. Electron. Agric. 184:106067. doi: 10.1016/j.compag.2021.106067

[ref11] Huang H. Lan Y. Deng J. Yang A. Deng X. Zhang L.. (2018). A semantic labeling approach for accurate weed mapping of high resolution UAV imagery. Sensors 18:2113. doi: 10.3390/s18072113, PMID: 29966392 PMC6069478

[ref12] Hung W. C. Tsai Y. H. Liou Y. T. Lin Y. Y. Yang M. H. (2018). Adversarial learning for semi-supervised semantic segmentation. arXiv [Preprint] arXiv:1802.07934.

[ref13] Ioffe S. Szegedy C. (2015). Batch normalization: accelerating deep network training by reducing internal covariate shift. Int. Conf. Mach. Learn. 37, 448–456.

[ref14] Islam N. Rashid M. M. Wibowo S. Xu C. Y. Morshed A. Wasimi S. A.. (2021). Early weed detection using image processing and machine learning techniques in an Australian chilli farm. Agriculture 11:387. doi: 10.3390/agriculture11050387

[ref15] Jiang H. Zhang C. Qiao Y. Zhang Z. Zhang W. Song C. (2020). CNN feature based graph convolutional network for weed and crop recognition in smart farming. Comput. Electron. Agric. 174:105450. doi: 10.1016/j.compag.2020.105450

[ref16] Khan S. Tufail M. Khan M. T. Khan Z. A. Iqbal J. Alam M. (2021). A novel semi-supervised framework for UAV based crop/weed classification. PLoS One 16:e0251008. doi: 10.1371/journal.pone.0251008, PMID: 33970938 PMC8109769

[ref17] Kudsk P. Streibig J. C. (2003). Herbicides–a two-edged sword. Weed Res. 43, 90–102. doi: 10.1046/j.1365-3180.2003.00328.x

[ref001] Lai X. Tian Z. Jiang L. Liu S. Zhao H. Wang L.. (2021). Semi-supervised semantic segmentation with directional context-aware consistency. In Proceedings of the IEEE/CVF Conference on Computer Vision and Pattern Recognition, 1205–1214.

[ref18] Liu B. Bruch R. (2020). Weed detection for selective spraying: a review. Curr. Robot. Rep. 1, 19–26. doi: 10.1007/s43154-020-00001-w

[ref19] Long J. Shelhamer E. Darrell T. (2015). Fully convolutional networks for semantic segmentation. Proc. IEEE Conf. Comput. Vis. Pattern Recognit. 79, 3431–3440. doi: 10.1109/CVPR.2015.7298965 27244717

[ref20] Lottes P. Khanna R. Pfeifer J. Siegwart R. Stachniss C. (2017). “UAV-based crop and weed classification for smart farming.” in *IEEE International Conference on Robotics & Automation*. Singapore: IEEE. July 21, 2017.

[ref21] Lottes P. Stachniss C. (2017). “Semi-supervised online visual crop and weed classification in precision farming exploiting plant arrangement.” in *2017 IEEE/RSJ International Conference on Intelligent Robots and Systems (IROS)*. September 24-28, 2017; 5155–5161.

[ref22] Ma X. Deng X. Qi L. Jiang Y. Li H. Wang Y.. (2019). Fully convolutional network for rice seedling and weed image segmentation at the seedling stage in paddy fields. PLoS One 14:e0215676. doi: 10.1371/journal.pone.0215676, PMID: 30998770 PMC6472823

[ref23] Nair V. Hinton G. E. (2010). “Rectified linear units improve restricted boltzmann machines.” in *International Conference on Machine Learning.* June 21, 2010.

[ref24] Pérez-Ortiz M. Peña J. M. Gutiérrez P. A. Torres-Sánchez J. Hervás-Martínez C. López-Granados F. (2015). A semi-supervised system for weed mapping in sunflower crops using unmanned aerial vehicles and a crop row detection method. Appl. Soft Comput. 37, 533–544. doi: 10.1016/j.asoc.2015.08.027

[ref25] Rakhmatulin I. Kamilaris A. Andreasen C. (2021). Deep neural networks to detect weeds from crops in agricultural environments in real-time: a review. Remote Sens. (Basel) 13:4486. doi: 10.3390/rs13214486

[ref26] Ramirez W. Achanccaray P. Mendoza L. Pacheco M. (2020). “Deep convolutional neural networks for weed detection in agricultural crops using optical aerial images.” in *Proceedings of the 2020 IEEE Latin American GRSS & ISPRS Remote Sensing Conference (LAGIRS)*. March 22–26, 2020. Santiago, Chile, 133–137.

[ref27] Ronneberger O. Fischer P. Brox T., (2015). “U-net: convolutional networks for biomedical image segmentation” in: *International Conference on Medical Image Computing and Computer-Assisted Intervention*. November 18, 2015; 234–241.

[ref28] Sa I. Popović M. Khanna R. Chen Z. Lottes P. Liebisch F.. (2018). WeedMap: a large-scale semantic weed mapping framework using aerial multispectral imaging and deep neural network for precision farming[J]. Remote Sens. (Basel) 10:1423. doi: 10.3390/rs10091423

[ref29] Shrivastava A. Gupta A. Girshick R. (2016). “Training region-based object detectors with online hard example mining.” in *Proceedings of the IEEE Conference on Computer Vision and Pattern Recognition*. June 26-July 1, 2016; 761–769.

[ref30] Yang L. Zhuo W. Qi L. Shi Y. Gao Y. (2021). ST++: make self-training work better for semi-supervised semantic segmentation. arXiv [Preprint arXiv:2106.05095].

[ref31] You J. Liu W. Lee J. (2020). A DNN based semantic segmentation for detecting weed and crop. Comput. Electron. Agric. 178:105750. doi: 10.1016/j.compag.2020.105750

[ref32] Zhao H. Shi J. Qi X. Wang X. Jia J., (2017). Pyramid scene parsing network, in *Proceedings of the IEEE Conference on Computer Visuion Pattern Recognition*. July 22-July 25, 2017; 2881–2890.

[ref33] Zhu J. Y. Park T. Isola P. Efros A. A. (2017). “Unpaired image-to-image translation using cycle-consistent adversarial networks.” in *Proceedings of the IEEE International Conference on Computer Vision*. October 22-29, 2017; 2223–2232.

